# CYP1B1 enhances the resistance of epithelial ovarian cancer cells to paclitaxel *in vivo* and *in vitro*

**DOI:** 10.3892/ijmm.2014.2041

**Published:** 2014-12-16

**Authors:** ZHUANGYAN ZHU, YAQIN MU, CAIXIA QI, JIAN WANG, GUOPING XI, JUNCHENG GUO, RUORAN MI, FUXI ZHAO

**Affiliations:** 1 Department of Obstetrics and Gynecology, Shanxi Datong University School of Medicine, Datong, Shanxi 037009, P.R. China; 2 Institute of Immunology, Shanxi Datong University School of Medicine, Datong, Shanxi 037009, P.R. China; 3 Department of Obstetrics and Gynecology, Tianjin Medical University General Hospital, Tianjin 300052, P.R. China

**Keywords:** epithelial ovarian cancer, cytochrome P450 1B1, paclitaxel, drug resistance, α-naphthoflavone

## Abstract

Ovarian cancer (OC) is the most frequent cause of mortality among gynecological malignancies, with a 5-year survival rate of approximately 30%. The standard regimen for OC therapy includes a platinum agent combined with a taxane, to which the patients frequently acquire resistance. Resistance arises from the oxidation of anticancer drugs by CYP1B1, a cytochrome P450 enzyme overexpressed in malignant OC. The aim of the present study was to determine the role of CYP1B1 expression in the drug resistance of OC to the taxane, paclitaxel (PTX). Immunohistochemical staining was used to assess CYP1B1 expression in a panel of ovarian samples (53 primary cancer samples, 14 samples of metastastic cancer, 30 benign tumor samples and 19 normal tissue samples). Semi-quantitative RT-PCR was also performed to determine CYP1B1 expression in several OC cell lines. Finally, we used proliferation and toxicity assays, as well as a mouse xenograft model using nude mice to determine whether α-naphthoflavone (ANF), a CYP1B1 specific inhibitor, reduces resistance to PTX. CYP1B1 was overexpressed in the samples from primary and metastatic loci of epithelial ovarian cancers. In some cell lines, PTX induced CYP1B1 expression, which resulted in drug resistance. Exposure to ANF reduced drug resistance and enhanced the sensitivity of OC cells to PTX *in vitro* and *in vivo*. The expression profile of CYP1B1 suggests that it has the potential to be a useful diagnostic marker and prognostic factor for malignant OC. The inhibition of CYP1B1 expression by specific agents may provide a novel therapeutic strategy for the treatment of patients resistant to PTX and may improve the prognosis of these patients.

## Introduction

Ovarian cancer (OC) is the most common cause of mortality among gynecological malignancies. Epithelial OC accounts for approximately 85–90% of primary OC cases (1). Despite the development in surgical technologies and adjuvant chemotherapy using platinum-based drugs in combination with taxanes, the 5-year survival rate for patients with the disease still remains at 30%. The poor prognosis is mainly due to the late presentation of symptoms or only apparent syndrome during the metastasis of the disease. In addition, OC has an unpredictable response to chemotherapy as an outcome of intrinsic or acquired drug resistance (2). Therefore, there is an urgent need for the identification of novel prognostic risk factors and for the development of novel therapeutic strategies for malignant OC. In addition, the further elucidation of the mechanisms underlying the chemotherapeutic resistance is required.

There are three major milestones in OC chemotherapy: drug development, including alkylating agents in the 1970s, cisplatin drugs in the 1980s and paclitaxel (PTX, taxol) in the 1990s. Paclitaxel is a natural product with anticancer activity obtained through a semi-synthetic process from *Taxus baccata*. Due to no cross-resistance with platinum-based drugs, it has become the first-line drug and by far the most effective chemotherapeutic agent for the treatment of OC. However, the application of PTX has been progressively limited in recent years mainly due to the occurrence of drug resistance and adverse complications which also diminish its therapeutic effects in OC (3–5).

The cytochrome P450 (CYP) enzymes are a family of important hemoprotein monooxygenases that catalyze the oxidation of a wide range of endogenous and exogenous xenobiotics, such as anticancer drugs, which results in drug degradation and inactivation (6,7). A number of cytochrome P450 family members can interfere with the metabolism of a range of anticancer drugs, such as PTX, docetaxel (DTX) and cyclophosphamide, which have been used in the chemotherapy of various cancers, including OC (8,9). These enzymes have cell-or tissue-specific expression, while some of them, particularly CYP1B1, are overexpressed in a wide range of cancers (10–15). The overexpression of CYP1B1 proteins in cancer cells may affect their sensitivity in reacting to anticancer drugs. *In vitro* studies have indicated that CYP1B1 increases the drug resistance of cells exposed to DTX and antagonizes the anticancer effects of DTX (16). However, to the best of our knowledge, reports on whether CYP1B1 mediates resistance to PTX in OC chemotherapy are limited.

In the present study, we investigated the expression profile of CYP1B1 in samples from patients with OC and confirmed its high expression in malignant cases compared to benign cases and normal ovarian tissue. In PTX-sensitive and -resistant cell lines, we identified the link between PTX-induced CYP1B1 expression and resistance to PTX. A specific inhibitor of CYP1B1, α-naphthoflavone (ANF), reversed the resistance to PTX and recovered the sensitivity of OC cells in response to PTX *in vitro* and *in vivo*. These findings are of great importance in terms of identifying novel diagnostic markers and prognostic factors for OC. Furthermore, our data provide insight into the development of potential therapeutic strategies for the treatment of paclitaxel-resistant OC patients.

## Materials and methods

### Patient samples and exclusion criteria

All 53 paraffin-embedded tissue blocks from patients with OC were provided by the Department of Pathology, Tianjin Medical University General Hospital, Tianjin, China. None of the patients had been previously treated or had received chemotherapy prior to surgery. The general information of the patients, the FIGO stage and pathological stage, as well as the histopathological characteristics of the patients are presented in Table I. Other tissue samples were comprised of 14 metastatic samples from the 53 patients with OC, 30 benign ovarian tumor samples and 19 normal ovarian tissue samples. The control group and the OC group were designed and selected with strict matching criteria so that there were no differences in age, family history or body mass index of the patients.

### Immunohistochemistry

Paraffin-embedded sections of normal ovaries and ovarian cancers were cut 4–5 *μ*m in size. To establish the distribution, intensity and cellular localization of CYP1B1, one section from each sample was counterstained with haematoxylin and eosin (H&E) and mounted using standard protocols. All other sections were dewaxed, rehydrated and washed in PBS, then immunostained with a polyclonal rabbit anti-human CYP1B1 antibody (CYP1B11-A; Alpha Diagnostic International, San Antonio, TX, USA) following the SP method. The immunohistochemical detection of CYP1B1 was performed using a DAB kit (Beijing Zhongshan Biotechnology Co., Beijing, China) to amplify the signals following the manufacturer’s instructions. CYP1B1 immunostaining was regarded as positive with brown granules exhibited in the cytoplasm of a cell. The overall immunoreactivity was assessed according to the percentage of positive cells in each sample as either strong (>50%), moderate (5–50%) or negative (<50%). Breast cancer sections previously diagnosed as CYP1B1-positive were used as the positive control, and samples stained with PBS only were used as the negative control.

### Cell culture and MTT assay

The COC1 cell line was derived from the ascites of patients with poorly differentiated OC provided by the Institute of Hematology, Chinese Academy of Medical Sciences, Tianjin, China. The HO-8910 and HO-8910PM cell lines obtained from the Shanghai Institute of Cell Biology, Chinese Academy of Sciences were both derived from human ovarian serous adenocarcinomas, yet, the latter had greater invasive and metastatic potential. The A2780 human ovarian epithelial cancer cell line established by the Cancer Institute of Chinese Academy of Medical Sciences is sensitive to PTX treatment. The A2780TS PTX-resistant cell line was established at the Tumor Hospital of Guangxi Medical University, Nanning, China and was derived by the continuous culture of A2780 cells with increasing amounts of PTX. All cell lines were cultured in RPMI-1640 + 10% fetal bovine serum (Gibco, Carlsbad, CA, USA) with 100 U/ml of penicillin-streptomycin. Paclitaxel was stored at a concentration of 30 mg/ml in saline and was further diluted to a final concentration of 1 mg/ml prior to use.

For MTT assay, 2×10^4^ cells/well were seeded in a 96-well plate for overnight growth and then treated with PTX at various concentrations for a further 24, 48 or 72 h. The cells were then incubated with 20 liters of 5 mg/ml MTT for 4 h at 37°C followed by the measurement of absorbance at 490 nm using a microplate reader (Thermo Varioskan Flash 4; Thermo Fisher scientific, Inc., Waltham, MA, USA), which indirectly reflected the proliferation rate of the cells. All samples were analyzed in triplicate and all experiments were repeated 3 times. The data are presented as the means ± standard deviation.

### RT-PCR

The cells were trypsinized and pelleted following centrifugation. The cell pellets were lysed in TRIZol reagent (Invitrogen) and total RNA was purified by chloroform-isopropanol extraction and subsequently resuspended in DEPC H_2_O. First-strand cDNA was synthesized using a standard protocol and 3.0 l of the synthesized cDNA product was used in the following PCR reactions. PCR amplifications of CYP1B1 cDNA generated a product of 380 bp using the forward primer, 5′-CACTGCCAACACCTCTGTCTT-3′ and the reverse primer, 5′-CAAGGAGCTCCATGGACTCT-3′. The product of MDR-1 was 650 bp with the forward primer, 5′-ACACCCGA CTTACAGATGATGT-3′ and the reverse primer, 5′-CGAGATGGGTAACTGAAGTGAA-3′. The PCR products were analyzed by DNA gel electrophoresis on a 1% agarose gel and the intensity of each band was quantified using Bandscan software. The relative expression levels of CYP1B1 and MDR-1 were normalized to the intensity of the β-actin bands, which served as a loading control; data are presented as the means ± standard deviation.

### Western blot analysis

For western blot analysis, 5–10×10^6^ cells were sonicated in lysis buffer (50 mM Tris-HCL pH 7.4, 150 mM NaCl, 1 mM EDTA, 1% Triton-X 100, 1% sodium deoxycholate, 0.1% SDS, 1 mM PMSF, 50 *μ*g/ml aprotinin and 50 *μ*g/ml leupeptin). The cell lysates were centrifuged at 12,000 rpm for 15 min at 4°C. The supernatant was collected and analyzed by sodium dodecyl sulfate polyacrylamide gel electrophoresis (SDS-PAGE). Proteins were transferred from the SDS-PAGE gel to the nitrocellulose membrane using the wet transfer method. Western blot anlaysis was conducted using anti-CYP1B1 primary antibody (1:500 dilution) and HRP-conjugated secondary antibody. The blots were visualized using a DAB kit following the manufacturer’s instructions.

### Animal experiments

BALB/c nude mice were purchased from the China Pharmaceutical and Biological Products Laboratory Animal Center, Beijing, China and maintained under specific pathogen-free (SPF) conditions in the research animal facilities of our institute. All animal procedures were performed according to Chinese animal welfare legislation.

A total of 18 female, 5–7-week-old, 18–20 g in weight nude mice were sublethally irradiated at 300 cGy 24 h prior to the injection of the cancer cells. Following local sterilization, 2×10^7^ A2780TS cells were injected subcutaneously into the subscapular region of the irradiated nude mice. After 3 weeks, when the subcutaneous tumor xenografts had reached approximately 10 mm^3^ in volume, these mice were divided randomly into 3 groups and were injected intraperitoneally with saline, 3 mg/kg PTX or 3 mg/kg PTX + 20 mg/kg ANF once every 4 days. All the mice were sacrificed on day 20 after the first treatment. The mice were sacrificed after anesthesia. The size of the tumors was measured and the tumor morphology was analyzed histochemically by H&E staining. The tumor growth inhibition rate was calculated and data are presented as the means ± standard deviation.

### Flow cytometric analysis

The A2780TS cells in the logarithmic growth phase were incubated with ANF at various concentrations in the presence or absence of 20 mg/ml of PTX for 24 h at 37°C. Untreated cells were used as the negative controls. The cells were fixed in 70% ethanol for 18–24 h at 4°C. Flow cytometric analysis was performed following propidium iodide (PI) staining in the dark for 30 min.

### Statistical analysis

The data were analyzed using the Student’s t-test, single factor analysis of variance (ANOVA) or Pearson’s correlation analysis using SPSS 11.5 software (SPSS Inc. Chicago, IL, USA). Differences were considered as statistically significant with a value of P<0.05.

## Results

### CYP1B1 is highly expressed in epithelial OC

In primary OC, several cytochrome P450 enzymes, most notably CYP1B1, are overexpressed at a significantly higher level compared with normal ovaries (17). Thus, CYP1B1 is regarded as a novel target for anticancer therapy. To verify the expression levels of CYP1B1 in the OC samples, we performed immunohistological analysis of the paraffin-embedded samples obtained from the 53 patients with OC at our hospital who had been diagnosed as epithelial OC at various clinical and pathological stages (Table I).

**Figure 1:**
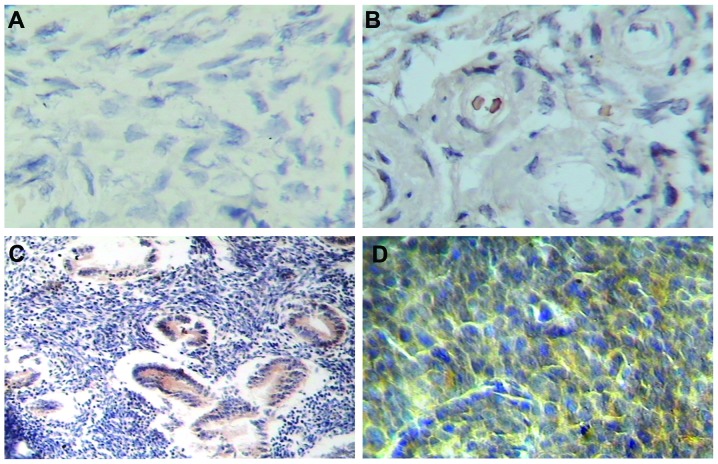
Expression of CYP1B1 in ovarian cancer, benign ovarian tumor and normal ovarian tissue samples. (A) Negative expression of CYP1B1 in normal ovarian tissue. (B) Expression of CYP1B1 in benign ovarian tumors. (C) Positive expression of CYP1B1 in ovarian cancer. (D) Positive expression of CYP1B1 in metastatic ovarian cancer.

CYP1B1 was generally presented immunohistologically as brownish yellow patches or diffuse areas in the cytoplasm of the OC cells. We observed negative CYP1B1 expression in all the normal ovarian tissue samples, yet positive immunoreactivity in 13.33% of the benign ovarian tumor samples (Fig. 1A and B). On the contrary, outstanding positive CYP1B1 immunoreactivity was observed in 49 of the 53 (92.45%) samples from the patients with OC. More importantly, the metastatic samples displayed a similar pattern of CYP1B1 expression in 13 out of the 14 samples (92.86%) (Fig. 1C and D).

These expression levels were significantly higher than those observed in benign ovarian tumor or normal ovarian tissue samples (χ^2^=51.96; P<0.01). Compared to the samples from the patients with maglinant OC in the earlier clinical stages (I and II), a higher expression of CYP1B1 was observed in the samples obtained from patients with OC in the later clinical stages (III and IV) (χ^2^=4.40, P<0.05) (Table I).

### PTX induces CYP1B1 expression in OC cell lines

**Figure 2:**
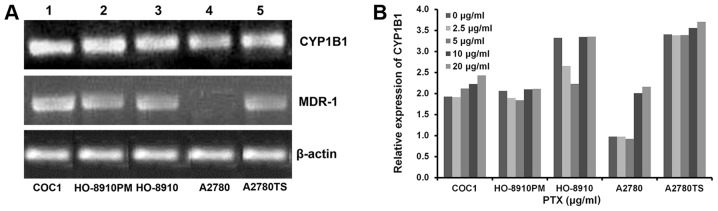
Paclitaxel (PTX)-induced CYP1B1 expression in ovarian cancer cell lines. (A) RT-PCR of total mRNA or mRNA levels of CYP1B1, MDR-1 and β-actin (as a loading control) in epithelial ovarian cancer cell lines (COC1, HO-8910, HO-8910PM, A2780 and A2780TS) as indicated. (B) The COC1, HO-8910, HO-8910PM, A2780 and A2780TS ovarian cancer cell lines were cultured and treated with various concentrations (0, 2.5, 5, 10 and 20 *μ*g/ml) of PTX for 24 h before harvesting. The leftmost column in each group represents the lowest concentration of PTX (0 *μ*g/ml), while the rightmost column in each group represents the highest concentration of PTX (20 *μ*g/ml). From left to right, the intervening columns represent 2.5, 5 and 10 *μ*g/ml of PTX. The mRNA expression level of CYP1B1 was measured by RT-PCR. The relative expression was calculated by normalization to the mRNA level of β-actin. The data are presented as the means ± standard deviation.

PTX is one of the natural broad-spectrum antitumor drugs that is used as first-line chemotherapy in OC therapy. However, the application of PTX and its anticancer effects have been diminished due to the increasing incidence of drug resistance or cross resistance. To evaluate the role of CYP1B1 in resistance to PTX, we measured the mRNA levels of CYP1B1 in several OC cell lines, including COC1, HO-8910PM, A2780 and A2780TS cells. As a positive control, the expression of the drug-resistant gene, MDR-1, was also analyzed and its expression was not observed in the PTX-sensitive cell line, A2780, but was observed in the PTX-resistant cell line, A2780TS. By contrast, the baseline mRNA levels of CYP1B1 were very similar in the A2780 and A2780TS cells with only a slight difference between each other (Fig. 2A). Nevertheless, the degree of difference in the relative CYP1B1 mRNA levels of these 2 cell lines became more evident following normalization to β-actin mRNA levels, which served as a loading control (Fig. 2B).

To determine whether CYP1B1 plays a role in resistance to PTX in OC, we incubated various OC cell lines with increasing amounts of PTX for 24 h and measured the mRNA levels of CYP1B1 after harvesting. The results revealed that in the PTX-resistant cell line, A2780TS, the relatively high baseline expression levels of CYP1B1 remained constant with only slight changes with the increasing amounts of PTX. Similar results were also obtained with the COC1, HO-8910PM and HO-8910 cell lines, which showed no or only slight changes in CYP1B1 expression during treatment with PTX. On the contrary, in the PTX-sensitive A2780 cell line, even though the baseline levels of CYP1B1 were relatively low, its expression increased substantially in the presence of PTX at concentrations over 10 g/ml (Fig. 2B). These results suggest that PTX increases CYP1B1 expression in A2780 cells and that PTX is more effective in OC cells with low or negative CYP1B1 expression.

### CYP1B1 inhibition enhances the sensitivity of A2780TS cells in response to PTX

**Figure 3:**
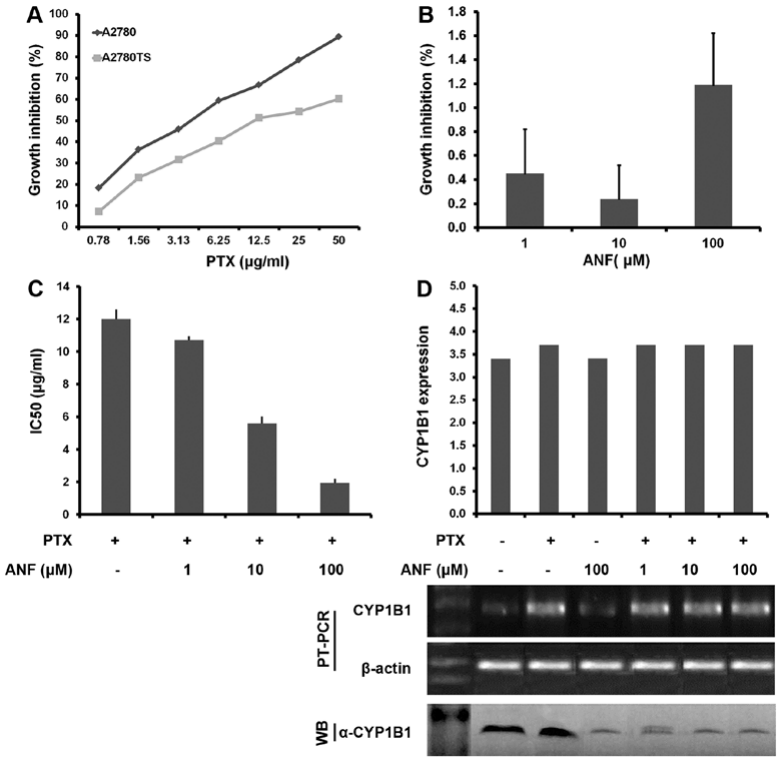
Treatment with α-naphthoflavone (ANF) reverses resistance to paclitaxel (PTX) in A2780TS cells. (A) Growth inhibition of A2780 and A2780TS cells following treatment with <1–50 mg/ml (PTX) for 72 h. (B) Growth inhibitory effects on A2780TS cells in the presence of titrated concentrations (1, 10 or 100 *μ*M) of ANF. (C and D) Effects of combined treatment with 20 *μ*g/ml PTX and 1, 10 or 100 *μ*M ANF on A2780TS cells. (C) Effects of combined PTX and ANF treatment on the growth (IC50) of A2780TS cells. (D) Effects of combined treatment with PTX and ANF or 100 *μ*M ANF alone on the mRNA and protein expression of CYP1B1, as assessed by RT-PCR and western blot analysis (WB), respectively.

Compared to the A2780 cells, the A2780TS cells had relatively higher levels of CYP1B1 expression which may result from previous exposure to PTX. To investigate this hypothesis, we treated these PTX-sensitive and PTX-resistant cells with serially diluted PTX at concentrations <1–50 *μ*g/ml for 72 h. The growth of the A2780 cells was inhibited by treatment with increasing amounts of PTX with the highest inhibition rate being almost 90%. This inhibitory effect was also observed in the PTX-resistant A2780TS cells, although to a much lesser extent (Fig. 3A). These results confirmed that the A2780TS cells were more resistant to PTX treatment than the A2780 cells and express the highest levels of CYP1B1.

ANF is a potent inhibitor of cytochrome P450 CYP1 family members, including CYP1A1, CYP1A2 and CYP1B1 (18). Thus, we assessed the effects of CYP1B1 inhibition on PTX-resistant A2780TS cells. When increasing the concentration of ANF (from 1 to 100 *μ*M), no significant inhibitory effect was observed on the growth of A2780TS cells (cell growth inhibition only increased from 0.45±0.37% at 1 *μ*M to 1.19±0.43% at 100 *μ*M; Fig. 3B). However, the growth of the same cells was significantly decreased by ANF in a dose-dependant manner in the presence of PTX which was indicated by reduced IC50 values (Fig. 3C). At the highest concentration of ANF, the IC50 value of the A2780TS cells in response to PTX was even lower than the reactivity of the A2780 PTX-sensitive cells (data not shown). These data indicate that the inhibition of CYP1B1 enhances the sensitivity of A2780TS cells to PTX. To evaluate the status of CYP1B1 under these conditions, we performed RT-PCR and western blot analysis which confirmed the reduced protein levels (Fig. 3D, bottom panel), but not the mRNA levels (Fig. 3D, top panel) of CYP1B1 in the presence of both PTX and ANF. These results suggest that CYP1B1 plays a role in the resistance of A2780TS cells to PTX and that the inhibition of CYP1B1 protein expression (but not mRNA expression) by ANF enhances the sensitivity of the cells to PTX.

### Concurrent PTX therapy and inhibition of CYP1B1 induce apoptosis and interfere with the cell cycle of A2780TS cells

**Figure 4:**
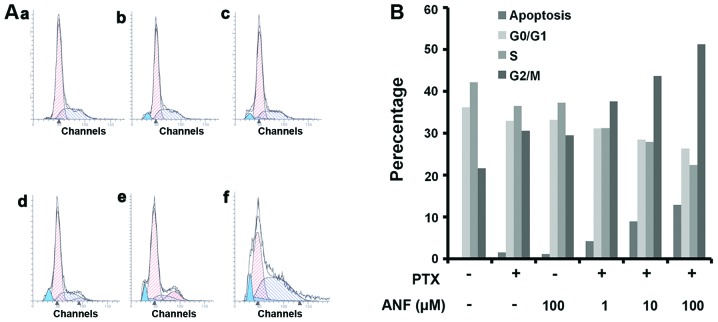
Analysis of the cell cycle in A2780TS cells treated with paclitaxel (PTX), α-naphthoflavone (ANF) or PTX and ANF. (A) Flow cytometric analysis of untreated A2780TS cells or A2780TS cells treated with PTX, ANF or a combination of PTX and ANF. (a) Untreated cells; (b) cells treated with 20 *μ*g/ml PTX; (c) cells treated with 100 *μ*M ANF; (d) cells treated with 20 *μ*g/ml PTX + 1 *μ*M ANF; (e) cells treated with 20 *μ*g/ml PTX + 10 *μ*M ANF; (f) cells treated with 20 *μ*g/ml PTX + 100 *μ*M ANF. (B) Analysis of apoptosis and the cell cycle (G0/G1, S and G2/M phase) in untreated A2780TS cells and A2780TS cells treated with PTX (20 *μ*g/ml), ANF (100 *μ*M) or a combination of PTX and ANF (20 *μ*g/ml PTX + 1, 10 or 100 *μ*M ANF).

To investigate the mechanisms involved in the growth inhibitory effects on A2780TS cells induced by the ANF-mediated inhibition of CYP1B1 in combination with PTX, we performed flow cytometric analysis of the untreated and treated A2780TS cells. Compared to the untreated cells, only low levels of apoptosis were observed in the A2780TS cells treated with either PTX or ANF (1.56 and 1.15%, respectively) (Fig. 4). However, the levels of apoptosis increased in a dose-dependant manner in the cells treated with both ANF and PTX (Fig. 4). We also investigated the cell cycle status of the untreated and treated A2780TS cells and found an increased number of cells in the G2/M phase along with a decreased number of cells in the G0/G1 and S phase in the cells treated with high concentrations of ANF in the presence of PTX. These results indicate that inhibiting CYP1B1 while treating the cells with PTX induces the apoptosis of A2780TS cells and arrests the cells at mitosis in a dose-dependant manner.

### Inhibition of CYP1B1 enhances the sensitivity of OC cells to PTX in vivo

**Figure 5:**
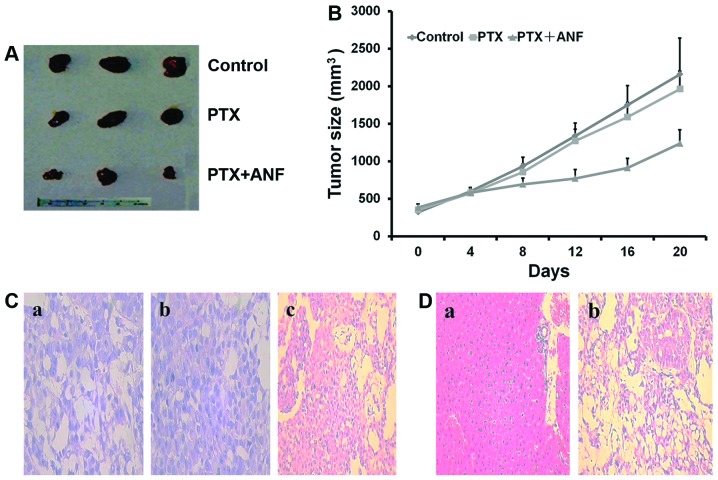
Treatment with α-naphthoflavone (ANF) antagonizes the resistance of ovarian cancer cells to paclitaxel (PTX) *in vivo*. (A) Images of representative tumors resulting from the subcutaneous injetion of A2780TS cells into nude BALB/c mice that were treated with saline (control, top row), 3 mg/kg PTX (middle row) or 3 mg/kg PTX and 20 mg/kg ANF (bottom row) when the tumors reached 10 mm^3^. (B) Tumor growth curves in nude mice with xenograft tumors treated with saline (control), PTX or PTX and ANF. (C) Images of hematoxylin and eosin-stained sections from representative tumors resulting from tumor xenografts in nude mice treated with (a) saline; (b) PTX; (c) PTX and ANF. (D) Representative images of hematoxylin and eosin-stained sections of (a) kidney and (b) liver from nude mice with xenograft tumors treated as described in (A) with PTX and ANF.

To verify the inhibitory effects of decreasing CYP1B1 activity in combination with PTX treatment on tumor growth *in vivo*, we established a tumor xenograft mouse model of OC through the subcutaneous injection of 2×10^7^ A2780TS cells into nude mice that had received sublethal irradiation. The xenotransplanted A2780TS cells formed solid tumors subcutaneously in the nude mice. These tumors had round, oval or irregular cauliflower-like shapes and showed similar patterns as human OC tumors within 3 weeks (Fig. 5A). When the tumors measured approximately 10 mm^3^, the mice were treated with PTX, PTX + ANF or saline only as a negative control. In the control group, the tumors developed rapidly with an almost 7-fold enlargement on day 20 after the first treatment. By contrast, the growth of the xenograft tumors was significantly reduced by combined treatment with PTX and ANF over a 20-day time course; however, this did not occur to such an extent in the mice treated with PTX only (Fig. 5A and B) (Table II). This confirmed that blocking CYP1B1 activity enhanced the resistance of OC cells to PTX *in vivo*.

Histopathological analysis of the saline-treated xenograft tumors displayed nested or irregularly clustered cancer cells with deeply stained atypical or lobulated nuclei and a large number of vacuoles in the cytoplasm (Fig. 5C, panel a). The proliferation of thin-walled blood vessels between cell clusters was visualized, as well as tumor necrosis in some areas of the xenografts. The morphology of the xenograft tumors in the PTX-treated group displayed similar patterns as the saline group (Fig. 5C, panel b). Nevertheless, the morphology of the tumors in the mice treated with ANF and PTX differed greately, with a substantially reduced number of cancer cells and less necrotic regions (Fig. 5C, panel c). We also evaluated the toxicity of different regimens. The post-treatment body weight change was regarded as an indirect indicator of drug toxicity. In the present study, all mice had a relative body weight (RBW) above 0.8 and no significant differences were observed before and after treatment in each group (P>0.05) (Table III). We also analyzed the histology of the kidneys and livers of the mice with xenograft tumors, which displayed no obvious pathological changes (Fig. 5D). Taken together, these data suggest that the combined PTX and ANF regimen causes no obvious toxicity.

## Discussion

It has been reported that a variety of cytochrome P450 enzymes, such as CYP1B1, CYP2A/2B and CYP2F1 are overexpressed in primary OC in comparison to normal ovarian tissue (17). Among these enzymes, CYP1B1 has been shown to have 92% positive immunoreactivity in 172 OCs equally distributed in both primary cancer foci and also metastatic tissue (19). In the present study, we confirmed the high CYP1B1 immunoreactivity in 53 primary samples and 14 metastatic tissue samples from patients with malignant epithelial OC and only in a small portion of benign tumors, which was consistent with previous reports. In addition, we further demonstrated that the CYP1B1 expression level was markedly higher in patients with OC in the late clinical stages (III and IV) than those with OC in the early stages, as classified by the FIGO staging system. These findings have several meanings. On the one hand, due to its specific expression pattern, CYP1B1 has the potential to be used as a diagnostic marker and prognostic factor for malignant OC (20,21). On the other hand, it provides new insight for the development of novel therapeutic strategies, such as CYP1B1-targeted inhibitors or T-cell based therapy for OC treatment.

PTX is a natural anticancer drug that is able to induce apoptosis in a variety of cancers and is particularly effective in OC therapy. CYP1B1 has been found to have an effect on tumor response to anticancer drugs, such as cyclophosphamide, PTX and DTX, which consequently affects pharmacokinetics and the therapeutic efficacy of the drugs (8,22,23). Rochat (24) conducted a retrospective study to investigate the corresponding mechanisms and found that CYP1B1 accelerated the degradation of anticancer drugs in resistant target cells. However, to the best of our knowledge, studies on the involvement of CYP1B1 in resistance to PTX are limited. Instead, there were a few publications describing the effects of CYP1B1 on DTX. McFadyen *et a*l found that CYP1B1 inactivated DTX so as to increase the anti-DTX effects in cells expressing CYP1B1. In addition, patients with medium or strong immunoreactivity to CYP1B1 had poorer survival rates than those with negative or weak reactions in response to DTX only or DTX plus platinum treatment (16,19). In the present study, we observed a high baseline mRNA level of CYP1B1 in the A2780TS PTX-resistant cells which had the highest IC50 value in response to PTX among all the cell lines examined. In addition, PTX increased CYP1B1 expression in the PTX-sensitive A2780 cells which had the lowest IC50 value. These data illustrate a link between CYP1B1 expression and resistance to PTX. Moreover, the resistance existing in A2780TS cells was very likely reversed by PTX-induced CYP1B1 expression which in turn inactivated PTX. These data provided potential solutions to combat PTX-associated drug resistance in cancer treatment, such as PTX inhibitors.

The activity of metabolic enzymes, such as CYP1B1 may be inhibited by a variety of natural and synthetic compounds. Flavonoids are a class of yellow, insoluble polyphenolic compounds with a 2-phenyl-chromone core extracted from a variety number of natural plants. Among the various flavonoid compounds, ANF is one of the most specific inhibitors of CYP1B1. We took advantage of the inhibitory effects of ANF to investigate the effects of CYP1B1 inhibition in A2780TS OC cells, which are resistant to PTX and express high levels of CYP1B1. In this model, we found that the ANF-mediated inhibition of CYP1B1 significantly increased the sensitivity of A2780TS cells to PTX in a dose-dependant manner. This effect was not observed following treatment with ANF alone, but was observed in the cells treated with both ANF and PTX. This finding demonstrates that ANF functions by inhibiting PTX-induced CYP1B1 expression. This conclusion is also consistent with the study by McFadyen *et al* in which ANF in combination with DTX, yet not alone, markedly enhanced the cytotoxic effects on CYP1B1-expressing V79MZh1B1 cells (16). In the present study, we observed a decrease in the expression of CYP1B1 at the protein level, but not the mRNA level (Fig. 3), suggesting that ANF plays a role in the post-transcriptional regulation of CYP1B1 expression; this requires further investigation.

In the present study, we further analyzed the cell cycle events of PTX- and/or ANF-treated cells to illustrate the anticancer mechanisms. We found that treatment with either PTX or ANF alone only had mild effects in arresting cells at the G2/M phase. Nevertheless, the function of ANF in the retention of A2780TS cells at the G2/M phase was substantially magnified in the presence of PTX. The tubulin/microtubule system is a major target site of PTX, which can promote microtubule polymerization and inhibit microtubule degradation to arrest the cells in the G2/M phase (25,26). Several different scenarios may explain these results. First, the inhibition of CYP1B1 activity may increase the availability or efficacy of PTX in the cells, and thus increase the amount of cell cycle arrest due to the direct action of PTX on microtubules. Conflicting evidence exists to support this hypothesis. *In vitro* studies have demonstrated that liver CYP2C8 and CYP3A4 are known to metabolize PTX (23), and a polymorphism in the CYP1B1 gene that is significantly associated with progression-free survival is not associated with PTX clearance (27). However, Bournique and Lemarie (28) demonstrated that CYP1B1 binds to docetaxel without metabolizing it. If the same is true of PTX, then the availability of PTX would be effectively increased if CYP1B1 was inhibited. Second, blocking the activity of CYP1B1 may reduce the accumulation of carcinogenic substances, including those related to estrogen (29,30), and this alteration may function in concert with PTX to slow the growth of the cancer cells. Third, the decreased activity of CYP1B1 may directly or indirectly arrest cell cycle progression. Although this area of research is relatively undeveloped, certain data indicate that the knockdown or inhibition of CYP1B1 in endometrial or OC cell lines causes cell cycle arrest (31,32).

We also investigated the apoptotic effects of PTX treatment in combination with the inhibition of CYP1B1. Apoptosis is the process of programmed cell death that also plays critical roles in tumorigenesis. A large number of chemotherapeutic reagents induce apoptosis and the sensitivity of cancer cells in response to these apoptotic agents may be a key determinant for various chemotherapy outcomes. Apart form the effects on microtubule bundling and mitotic arrest, PTX can induce apoptosis through the NF-κB/IκB pathway to promote the nuclear translocation of NF-κB and its DNA binding activity (33). There is another mechanism involved in PTX-induced apoptosis which was through the promotion of TRAIL activity to induce the protein levels of cell death receptor 4 (34). In the present study, when combined with PTX, ANF greatly increased the apoptosis of A2780TS cells. However, there was only a mild increase in the number of apoptotic cells at the highest concentration of ANF compared to the untreated cells (1.15 vs. 0%). It is not entirely clear whether the inhibition of CYP1B1 by itself has the potential ability to induce the apoptosis of cancer cells; this requires further investigation.

The subcutaneous xenotransplantation of A2780TS OC cells in BALB/c nude mice can replicate the developmental process and the morphology of human OCs. It is an ideal model to evaluate anticancer effects of PTX and CYP1B1 inhibition *in vivo*. Compared to the tumor size in the control mice, the significant difference in tumor growth between the PTX-treated mice and PTX + ANF-treated mice further confirmed that decreasing the expression or activity of CYP1B1 can potentiate the anticancer abilities of PTX, provided that both treatments are concurrent (P<0.01). These data are consistent with the *in vitro* observations in the present study. Besides, the regimen of PTX + ANF was well tolerated by all xenograft recipients. This provides strong theoretic and practical basis for future clinical applications.

In conclusion, we demonstrated that CYP1B1 was overexpressed in human malignant OC samples and that its expression levels were significantly higher than those in benign ovarian tumors or normal ovarian tissue. PTX induces CYP1B1 expression in OC cells and this enhanced expression results in resistance to PTX. The resistance to PTX in A2780TS cells was reversed by the downregulation of CYP1B1 expression using the specific inhibitor ANF *in vitro* and *in vivo*. Thus, ANF or another inhibitor of CYP1B1, in combination with PTX can combat the resistance of OC cells to anticancer drugs. Our findings provide the theoretical basis for the development of novel strategies for the clinical treatment of OC to further improve prognosis.

## Figures and Tables

**Table I:** Risk factors of CYP1B1 expression in patients with epithelial ovarian cancer.

	n	CYP1B1 expression		
		Positive (%)	χ^2^	P-value
Malignant epithelial ovarian cancer	53	49 (92.45)		
Clinical stage				
I + II	17	15 (88.24)		
III + IV	36	34 (94.44)	4.40	<0.05
Pathologic stage				
Class G1–G2	18	16 (88.89)		
Class G3	35	33 (91.43)	0.49	>0.05
Benign epithelial ovarian tumor	30	4 (13.33)		
Normal ovary	19	0 (0.0)	51.96	<0.01

**Table II:** Tumor inhibition in treated and untreated nude mice with xenograft tumors.

Group	Tumor size (mm^3^, means ± SD)		Tumor inhibition (%)	t	P-value
	Pre-treatment	Post-treatment			
Control	321.65±39.74	2159.74±84.39	0		
PTX	352.11±29.74	1964.27±239.56	9.05	1.071	>0.05
PTX + ANF	384.62±46.59	1235.67±184.93	42.79	5.726	<0.01

**Table III:** Changes in body weight of the treated and untreated nude mice with xenograft tumors.

Group	Weight (g, means ± SD)		RBW	t	P-value
	Pre-treatment	Post-treatment			
Control	19.53±1.50	23.55±1.28	1.21	0.893	>0.05
PTX	20.12±1.84	22.71±1.66	1.13	1.222	>0.05
PTX+ANF	21.01±1.64	22.61±1.14	1.08		

RBW, relative body weight.
